# Study of some graph theoretical parameters for the structures of anticancer drugs

**DOI:** 10.1038/s41598-024-64086-5

**Published:** 2024-06-10

**Authors:** Ibtisam Masmali, Muhammad Azeem, Muhammad Kamran Jamil, Ali Ahmad, Ali N. A. Koam

**Affiliations:** 1 Department of Mathematics, College of Science, Jazan University, 45142 Jazan, Saudi Arabia; 2 Department of Mathematics, Riphah International University, Lahore, Pakistan; 3 Department of Computer Science, College of Engineering and Computer Science, Jazan University, Jazan, Saudi Arabia

**Keywords:** Biochemistry, Chemical biology, Computational biology and bioinformatics, Drug discovery, Chemistry, Mathematics and computing

## Abstract

Eigenvalues have great importance in the field of mathematics, and their relevance extends beyond this area to include several other disciplines such as economics, chemistry, and numerous fields. According to our study, eigenvalues are utilized in chemistry to express a chemical compound’s numerous physical properties as well as its energy form. It is important to get a comprehensive understanding of the interrelationship underlying mathematics and chemistry. The anti-bonding phase is correlated with positive eigenvalues, whereas the bonding level is connected with negative eigenvalues. Additionally, the non-bonded level corresponds to eigenvalues of zero. This study focuses on the analysis of various structures of anticancer drugs, specifically examining their characteristic polynomials, eigenvalues of the adjacency matrix, matching number and nullity. Consequently, the selected structures of the aforementioned anticancer drugs exhibit stability since they are composed of closed-shell molecules, characterized by a nullity value of zero.

## Introduction

Cancer is defined as the uncontrolled growth of abnormal cells in the human system over an extended period of time. Carcinogens are any substances that have the potential to cause cancer. One of the main elements of smoke from cigarettes is an organic compound known as a carcinogen. Carcinogens are known to cause cancer. It is able to travel to different parts of the body.

This problem manifests itself in a variety of ways, some of which include an abnormal bleeding, or lump, a persistent cough, and a loss of weight. Chewing tobacco, being overweight, having a bad diet, not exercising enough, and drinking too much alcohol are the primary factors that lead to this malignant illness. This life-threatening condition may be managed with a variety of therapeutic approaches, such as targeted therapy, hormone therapy, chemotherapy, radiation, surgery and many more. Medications classified as anticancer drugs, such as metabolites and alkylates are often administered in order to treat cancer, the illness^1–4^.

The field of science and mathematics is known as chemical graph theory which deals with chemical-based graphs, which are representations of molecular processes. The molecular graph theory makes it possible to define a wide variety of anticancer drug features^5,6^. In this work, many different medicational configurations are used, and they are measured using characteristics polynomials, adjacency matrix, eigenvalues, matching number and nullity. The nullity of a molecule is a significant contributor to its level of stability; hence, if the nullity of the molecule is zero, it is anticipated that the molecule will have a stable, closed-shell electron configuration. If nullity is greater than zero, the molecule is not just unstable but also very reactive, nonexistent, and has an open shell^7^. As a consequence of this, all of the structures of the anticancer drugs mentioned above are examples of closed-shell molecules that are stable due to the fact that their nullity is equal to zero.

### Literature review

There exists a substantial body of scholarly literature pertaining to the topics of characteristic polynomials, eigenvalues of adjacency matrices, matching numbers and nullity. This document presents a selection of current and significant papers, highlighting their respective value. The concept of nullity is quantified by the greatest degree of a vertex^8,9^. The literature contains scholarly works that go into the realm of pure mathematics and abstract theory on nullity, which may be found in the references^10–12^. The study conducted by^13^ explores unicyclic graphs in relation to their nullity and matching number. On the other hand,^14^ provides an analysis of bicyclic graphs. Additionally,^15^ investigates the line operation of unicyclic graphs and its impact on their nullity. These references provide valuable insights into the respective topics. The characterization of rank four graphs may be found in the work of^16^, while the characterization of rank five graphs is provided in the study of^17^ included in the references. To find upper limits on the nullity $n-2$ and $n-3,$ please refer to the work of^18^ and^19^ respectively. This study examines the nullity of a graph that has cut-points, as discussed in research paper^20^. The concept of trees is examined in relation to the nullity of a graph as presented in the work of^21^. The paper by^22^ presents a study on the correlation between the matching number and rank of a graph. The hypothesis about nullity is supported by a proof presented in the work of^23^.

In recent years, graph theory provide some efficient tools to study in different fields like in fuzzy^24,25^, in decision making^26–28^, in chemistry^29,30^, in molecular physics^31–33^. Particularly molecular insights into anti-Alzheimer’s drugs through predictive modeling using linear regression and QSPR analysis^34,35^. Supersaturation of a biopharmaceutical classification system^36–38^. Nanoscale covalent organic frameworks: from controlled synthesis to cancer therapy^39–41^. Recent advance in biological responsive nanomaterials for biosensing and molecular imaging application^42–44^. The ferroptosis signature predicts the prognosis and immune microenvironment of nasopharyngeal carcinoma^45–47^. Visualization of Zika virus infection via a light-initiated bio-orthogonal cycloaddition labeling strategy found in^48–50^.

### Preliminaries of the study

A molecular compound is often represented in a graphical format, where the individual constituents are displayed as vertices (which is also referred as nodes) and the connections between them are represented as edges (which is also referred as lines). In a similar vein, the chemical compounds being examined for their anticancer properties are subject to analysis, whereby several factors are evaluated. Graph theory offers many techniques, including Quantitative Structure-Activity Relationship (QSAR), Quantitative Structure-Property Relationship (QSPR), and Quantitative Structure-Toxicity Relationship (QSTR), which may be used by chemists and pharmacists to augment their research endeavors. In the field of mathematical chemical science, medicines are shown as molecular networks, whereby individual atoms are symbolized by vertices, and the connections between two atoms are denoted by edges^51,52^. Let us consider a molecular graph denoted as ${G}(\mathscr {V},{E}),$ where $\mathscr {V}$ represents the set of vertices and *E* represents the set of edges. We examine simple graphs with no production of cycles and many edges. Let $|\mathscr {V}\left( {G}\right) |=\mathscr {N}$ and $|{E}\left( {G}\right) |=\mathscr {M}$ denote the order and size of a graph *G*,  respectively. The adjacency matrix, represented as ${Bin}\left( {G}\right) ,$ is a mathematical representation of a graph. Its members indicate whether pairs of vertices in the graph are adjacent or not.The characteristic polynomial of a graph may be defined as the determinant of the matrix ${Bin}\left( {G}\right)$ minus ${\lambda }$ times the identity matrix *I*,  which is equal to zero. Here, *I* represents an identity matrix of the same order as the matrix *Bin*. The expression $\text {det}\left( {Bin}\left( {G}\right) -{\lambda }{I}\right) =0$ of the structure ${\lambda }$ gave some values in output which is symbolized as eigenvalues and we denoted those values as $\mathscr {E}\left( {CP}\left( {Bin}\left( {G}\right) ;{\lambda }\right) \right) .$ The mulitplicity of $\mathscr {E}\left( {CP}\left( {Bin}\left( {G}\right) ;{\lambda }\right) \right) =0$ is called as nullity of a network *G*,  and we symbolized this with the symbol ${Nul}\left( {G}\right)$^53^. The nullity of a bipartite network is described in^54^.

1 $$\begin{aligned} {Nul}\left( {G}\right) =\mathscr {N}-2\text {MN}\left( {G}\right) . \end{aligned}$$

**Figure 1:**
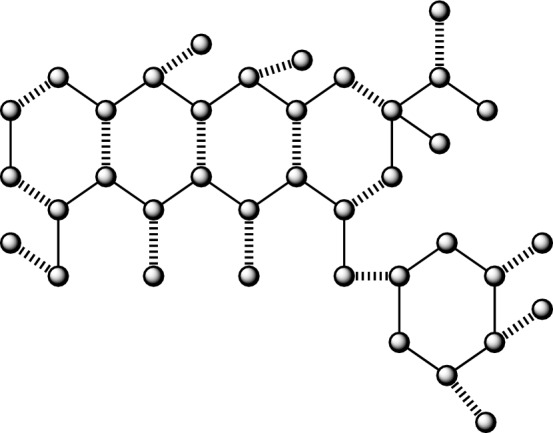
Anticancer drug structure Daunorubicin.

**Figure 2:**
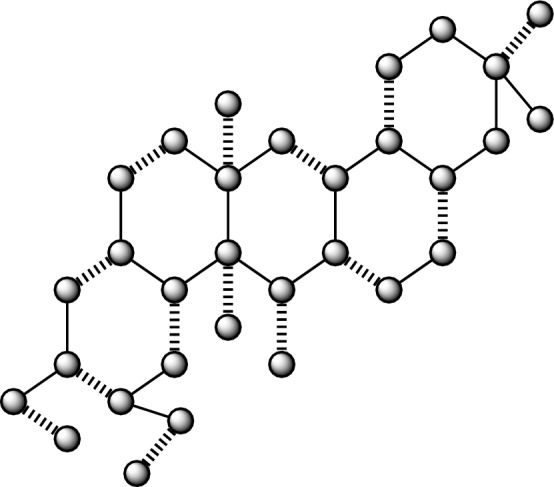
Anticancer drug structure Deguelin.

**Figure 3:**
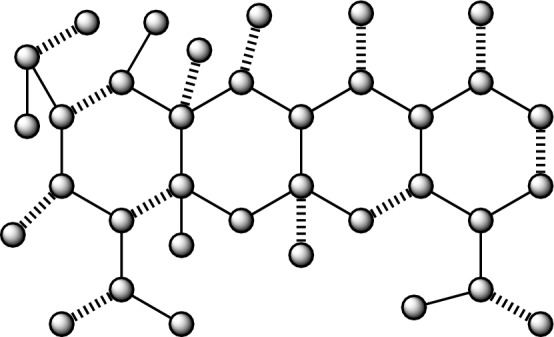
Anticancer drug structure Minocycline.

**Figure 4:**
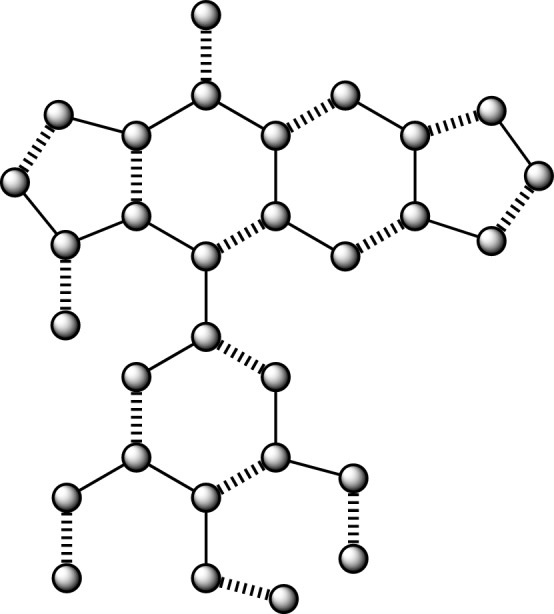
Anticancer drug structure Podophylotoxin.

**Figure 5:**
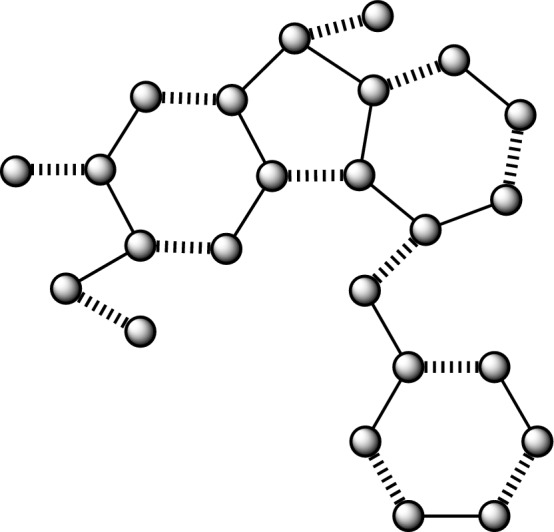
Anticancer drug structure Pterocellin-B.

**Figure 6:**
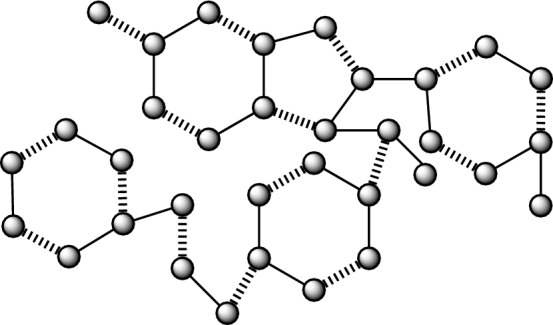
Anticancer drug structure Raloxifene.

The parameter $\text {MN}\left( {G}\right)$ in Eq. (1) is often referred to as the matching number. It is defined as the cardinality of the biggest maximum independent edge set, as stated in previous works^55,56^. In Figs. 1, 2, 3, 4, 5 and 6, the presence of wavy edges indicates that these edges are associated with the calculation of the matching number for all structures of anticancer drugs. Some graph theoretical study is conducted in^57–60^.

In the field of mathematics, the expression $\mathscr {E}=\sum _{{i}=1}^{k=|V\left( {G}\right) |}|{\lambda }_{i}|$ is presented as stated by^7^. The energy of a graph, denoted as *G*,  is defined as the absolute sum of all its eigenvalues. Whereas the negative inertia index is denoted by the quantity of negative eigenvalues *q*(*G*),  and the positive inertia index is denoted by the quantity of positive eigenvalues *p*(*G*).

### Main contribution of the study

Main contribution of this is mentioned in the following manner:

- This study focuses on the analysis of various structures of anticancer drugs
- Specifically examining their characteristic polynomials, eigenvalues of the adjacency matrix, matching number and nullity
- Consequently, the selected structures of the aforementioned anticancer drugs exhibit stability since they are composed of closed-shell molecules, characterized by a nullity value of zero.

## The findings pertaining to the correlation between the number and nullity of structures of anticancer drugs.

This research employs many medicinal structures, including the measurement of characteristic polynomials, eigenvalues of the adjacency matrix, matching number and nullity.

### The findings pertaining to the correlation between the number and nullity of the Daunorubicin anticancer medication structure

The graph derived from Daunorubicin has a vertex count of 38, denoted as $|\mathscr {V}\left( {G}_{1}\right) |,$ and a size is 42, denoted as $|\mathscr {E}\left( {G}_{1}\right) |.$ In addition, the node and lines set are formulated as

$$\begin{aligned} \mathscr {V}({G}_1)=&\{v_{i}:{i}=1,2,\dots ,38\},\\ {E}({{G}_1})=&\{{v}_{i}{v}_{{i}+1}:{i}=1,2,\dots ,17,29,30,\dots ,34\}\cup \{{v}_{1}{v}_{18},{v}_{3}{v}_{16},{v}_{5}{v}_{14},{v}_{7}{v}_{12},{v}_{9}{v}_{23},{v}_{9}{v}_{24},{v}_{21}{v}_{23},\\ {}&{v}_{22}{v}_{23},{v}_{4}{v}_{19},{v}_{6}{v}_{20},{v}_{17}{v}_{26},{v}_{25}{v}_{26},{v}_{15}{v}_{27},{v}_{13}{v}_{28},{v}_{11}{v}_{29},{v}_{30}{v}_{35},{v}_{32}{v}_{36},{v}_{33}{v}_{37},{v}_{34}{v}_{38}\}. \end{aligned}$$

Moreover, all these graphs detail can be found in the source^1^.

#### Lemma 1

If ${G}_1$ is derived from the molecular structure of the anticancer medication Daunorubicin, then ${Nul}\left( {G}_1\right) =0.$

#### Proof

It can be shown that ${G}_1$ represents the molecular structure of Daunorubicin, an anticancer medication. This structure is characterized by the presence of pendant vertices and a five-count of hexagons. By using the concept of nullity, we will ascertain the features polynomial ${CP}\left( {Bin}\left( {G}_1\right) ;{\lambda }\right) .$ The cost of the adjacency matrix, denoted as ${Bin}\left( {G}_1\right) ,$ is being considered. The graph ${G}_1$ is associated with a polynomial that represents its distinctive properties;

$${CP}\left( {Bin}\left( {G}_1\right) ;{\lambda }\right) =$$

$$\begin{aligned}&{\lambda }^{38} - 42{\lambda }^{36} + 795{\lambda }^{34} - 8988{\lambda }^{32} + 67817{\lambda }^{30} - 361464{\lambda }^{28} + 1405055{\lambda }^{26} - 4054216{\lambda }^{24} + 8754529{\lambda }^{22}\\ {}&- 14155626{\lambda }^{20} + 17032211{\lambda }^{18} - 15045768{\lambda }^{16} + 9546480{\lambda }^{14} - 4209628{\lambda }^{12} + 1228196{\lambda }^{10} - 219432{\lambda }^8\\ {}&+ 20816{\lambda }^6 - 736{\lambda }^4, \end{aligned}$$

By solving the equation ${CP}\left( {Bin}\left( {G}_1\right) ;{\lambda }\right) =0,$ we can determine the solution. For a given value of ${\lambda },$ the eigenvalues $\mathscr {E}\left( {CP}\left( {Bin}\left( {G}_1\right) ;{\lambda }\right) \right)$ of certain polynomials are calculated.

$$\mathscr {E}\left( {CP}\left( {Bin}\left( {G}_1\right) ;{\lambda }\right) \right) =$$

$$\begin{aligned}&\{-2.6239, -2.4161, -2.3057, -2.1135, -1.8746, -1.7229, -1.6579, -1.4520, -1.4142,\\ {}&-1.2734, -1.2248, -1, -0.9796, -0.6241, -0.5700, -0.5314, -0.2765, -5.1488e^{-16},\\ {}&-3.0326e^{-17}, 5.7596e^{-17}, 2.5391e^{-16}, 0.2765, 0.5314, 0.5700, 0.624, 0.9796, 0.9999,\\ {}&1.2248, 1.2734, 1.4142, 1.4520, 1.6579, 1.7229, 1.8746, 2.1135, 2.3057, 2.4161, 2.6240\}. \end{aligned}$$

It is observed that there is an absence of a precise zero value from the given data $\mathscr {E}\left( {CP}\left( {Bin}\left( {G}_1\right) ;{\lambda }\right) \right) =0$ and which is concluding that ${Nul}\left( {G}_1\right) =0.$

Moreover, as seen in Fig. 1, it is evident that there are a total of seventeen instances of wavy edges, which corresponds to the same numerical value ${M}\left( {G}_1\right)$ of graph ${G}_1.$

Moreover, its energy is 48.1216,  concluded by summing all the $\mathscr {E}\left( {CP}\left( {Bin}\left( {G}_1\right) ;{\lambda }\right) \right) .$

It can be inferred that the selected structure of the anticancer medicine is composed of stable, closed-shell molecules, as shown by its nullity value of zero. $\square$

### The findings pertaining to the correlation between the number and nullity of the Deguelin anticancer medication structure

A network resulting from Deguelin has a vertex count of 31, denoted as $|\mathscr {V}\left( {G}_{2}\right) |,$ and a size is 35, denoted as $|\mathscr {E}\left( {G}_{2}\right) |.$ Furthermore, the node and lines set have been established by

$$\begin{aligned} \mathscr {V}({G}_2)=&\{v_{i}:{i}=1,2,\dots ,31\},\\ {E}({{G}_2})=&\{{v}_{i}{v}_{{i}+1}:{i}=1,2,\dots ,21\}\cup \{{v}_{1}{v}_{22},{v}_{1}{v}_{24},{v}_{23}{v}_{24},{v}_{22}{v}_{26},{v}_{25}{v}_{26},{v}_{3}{v}_{20},{v}_{6}{v}_{19},{v}_{6}{v}_{27},\\ {}&{v}_{8}{v}_{17},{v}_{9}{v}_{14},{v}_{12}{v}_{28},{v}_{12}{v}_{29},{v}_{18}{v}_{30},{v}_{19}{v}_{31}\}. \end{aligned}$$

#### Lemma 2

If ${G}_2$ is derived from the molecular structure of the anticancer medication Deguelin, then ${Nul}\left( {G}_2\right) =0.$

#### Proof

It can be seen that ${G}_2$ represents the molecular structure of Deguelin, an anticancer medication, which is composed of pendant vertices and hexagons with a count of five. By using the concept of nullity, we will ascertain the features polynomial ${CP}\left( {Bin}\left( {G}_2\right) ;{\lambda }\right) .$The cost of the adjacency matrix, denoted as ${Bin}\left( {G}_2\right) ,$ is calculated.The graph ${G}_2$ is associated with a polynomial known as the characteristics polynomial.

$${CP}\left( {Bin}\left( {G}_2\right) ;{\lambda }\right) =$$

$$\begin{aligned}&{\lambda }^{31} - 35{\lambda }^{29} + 538{\lambda }^{27} - 4800{\lambda }^{25} + 27680{\lambda }^{23} - 108734{\lambda }^{21} + 298732{\lambda }^{19} - 579991{\lambda }^{17}\\ {}&+ 794418{\lambda }^{15} - 757870{\lambda }^{13} + 491005{\lambda }^{11} - 207099{\lambda }^9 + 52946{\lambda }^7 - 7159{\lambda }^5 + 364{\lambda }^3 \end{aligned}$$

By finding the solution to the equation ${CP}\left( {Bin}\left( {G}_2\right) ;{\lambda }\right) =0,$ we can determine the desired outcome. For a given value of ${\lambda },$ the eigenvalues $\mathscr {E}\left( {CP}\left( {Bin}\left( {G}_2\right) ;{\lambda }\right) \right)$ of certain polynomials are determined.

$$\mathscr {E}\left( {CP}\left( {Bin}\left( {G}_2\right) ;{\lambda }\right) \right) =$$

$$\begin{aligned}&\{-2.6647, -2.4060, -2.2066, -1.9562, -1.7068, -1.6021, -1.4394, -1.3540, -1.2439,\\ {}&-0.9619, -0.7974, -0.6577, -0.6136, -0.3360, -1.3965e^{-15}, -5.4120e^{-16}, -2.8286e^{-16},\\ {}&0.3360, 0.6136, 0.6577, 0.7974, 0.9619, 1.2439, 1.3539, 1.4394, 1.6021, 1.7068, 1.9562, 2.2066,\\ {}&2.4060, 2.6647\}. \end{aligned}$$

It is observed that there is an absence of a precise zero value from the given data $\mathscr {E}\left( {CP}\left( {Bin}\left( {G}_2\right) ;{\lambda }\right) \right) =0$ and which is concluding that ${Nul}\left( {G}_2\right) =0.$

Moreover, as seen in Fig. 2, it can be observed that there are a total of fourteen instances of wavy edges, which corresponds to the value of ${M}\left( {G}_2\right) .$

Furthermore, the energy of the system is determined to be 39.8922 by calculating the total of all the individual energies $\mathscr {E}\left( {CP}\left( {Bin}\left( {G}_2\right) ;{\lambda }\right) \right) .$

The determination has been made that the selected structure of the anticancer medicine is stable, consisting of closed-shell molecules, as shown by its nullity value of zero. $\square$

### The findings pertaining to the correlation between the number and nullity of the Minocycline anticancer medication structure

A network resulting from the application of Minocycline has a vertex count of 35, denoted as $|\mathscr {V}\left( {G}_{3}\right) |=35.$ The size of the graph, measured by the number of edges, is 38, represented as $|\mathscr {E}\left( {G}_{3}\right) |=38.$ In addition, the node and lines set have been established by.

$$\begin{aligned} \mathscr {V}({G}_3)=&\{v_{i}:{i}=1,2,\dots ,35\},\\ {E}({{G}_3})=&\{{v}_{i}{v}_{{i}+1}:{i}=1,2,\dots ,18\}\cup \{{v}_{1}{v}_{18},{v}_{1}{v}_{21},{v}_{20}{v}_{21},{v}_{21}{v}_{22},{v}_{2}{v}_{23},{v}_{3}{v}_{24},{v}_{3}{v}_{16},{v}_{4}{v}_{25},\\ {}&{v}_{5}{v}_{14},{v}_{7}{v}_{12},{v}_{8}{v}_{27},{v}_{11}{v}_{29},{v}_{28}{v}_{29},{v}_{29}{v}_{30},{v}_{14}{v}_{31},{v}_{16}{v}_{32},{v}_{17}{v}_{34},{v}_{33}{v}_{34},{v}_{34}{v}_{35}\}. \end{aligned}$$

#### Lemma 3

If ${G}_3$ is derived from the molecular structure of the anticancer medication Minocycline, then ${Nul}\left( {G}_3\right) =0.$

#### Proof

It can be seen that ${G}_3$ represents the molecular structure of Minocycline, an anticancer medication, which is composed of pendent vertices and a total of four hexagons. By using the concept of nullity, we will ascertain the features polynomial ${CP}\left( {Bin}\left( {G}_3\right) ;{\lambda }\right) .$The cost of the adjacency matrix, denoted as ${Bin}\left( {G}_3\right) ,$ is being considered.The graph ${G}_3$ is associated with a polynomial known as the characteristics polynomial.

$${CP}\left( {Bin}\left( {G}_3\right) ;{\lambda }\right) =$$

$$\begin{aligned}&{\lambda }^{35} - 38{\lambda }^{33} + 639{\lambda }^{31} - 6289{\lambda }^{29} + 40364{\lambda }^{27} - 178109{\lambda }^{25} + 554748{\lambda }^{23} - 1231634{\lambda }^{21}\\ {}&+ 1942413{\lambda }^{19} - 2138831{\lambda }^{17} + 1586669{\lambda }^{15} - 742058{\lambda }^{13} + 191923{\lambda }^{11} - 19807{\lambda }^9 \end{aligned}$$

By finding the solution to the equation ${CP}\left( {Bin}\left( {G}_3\right) ;{\lambda }\right) =0,$ we can determine the desired outcome. For a given value of ${\lambda },$ the eigenvalues $\mathscr {E}\left( {CP}\left( {Bin}\left( {G}_3\right) ;{\lambda }\right) \right)$ of certain polynomials are calculated.

$$\mathscr {E}\left( {CP}\left( {Bin}\left( {G}_3\right) ;{\lambda }\right) \right) =$$

$$\begin{aligned}&\{-2.7220, -2.4703, -2.1535, -1.958, -1.8610, -1.7995, -1.5414, -1.2942, -1.2650,\\ {}&-1.2180, -1.0589, -0.8945, -0.5091, -2.4486e^{-15}, -6.9816e^{-16}, -2.6909e^{-16}, -1.7541e^{-16},\\ {}&-7.2118e^{-17}, 3.9659e^{-17}, 1.1545e^{-16}, 2.4230e^{-16}, 3.2895e^{-16}, 0.5091, 0.894, 1.0589, 1.2180,\\ {}&1.2650, 1.2942, 1.5414, 1.7995, 1.8610, 1.9582, 2.1535, 2.4703, 2.7220\}. \end{aligned}$$

It is observed that there is an absence of a precise zero value from the given data $\mathscr {E}\left( {CP}\left( {Bin}\left( {G}_3\right) ;{\lambda }\right) \right) =0$ and which is concluding that ${Nul}\left( {G}_3\right) =0.$

Moreover, Fig. 3 depicts a total of thirteen instances of undulating boundaries, which corresponds to the value of ${M}\left( {G}_3\right) .$

Furthermore, the energy value is determined to be 41.4910 by aggregating all the $\mathscr {E}\left( {CP}\left( {Bin}\left( {G}_2\right) ;{\lambda }\right) \right)$ values.

The determination has been made that the selected structure of the anticancer medicine is stable, consisting of closed-shell molecules, as shown by its nullity value of zero. $\square$

### The findings pertaining to the correlation between the number and nullity of the Podophylotoxin anticancer medication structure

The graph derived from Podophylotoxin has an order of 30, denoted as $|\mathscr {V}\left( {G}_{4}\right) |,$ and size is 34, denoted as $|\mathscr {E}\left( {G}_{4}\right) |.$ Furthermore, the collection of vertices and edges is determined by

$$\begin{aligned} \mathscr {V}({G}_4)=&\{v_{i}:{i}=1,2,\dots ,30\},\\ {E}({{G}_4})=&\{{v}_{i}{v}_{{i}+1}:{i}=1,2,\dots ,16,19,20,\dots ,23\}\cup \{{v}_{1}{v}_{16},{v}_{16}{v}_{17},{v}_{3}{v}_{15},{v}_{4}{v}_{18},\\ {}&{v}_{5}{v}_{13},{v}_{7}{v}_{11},{v}_{14}{v}_{19},{v}_{19}{v}_{24},{v}_{21}{v}_{30},{v}_{29}{v}_{30},{v}_{22}{v}_{28},{v}_{27}{v}_{28},{v}_{23}{v}_{26},{v}_{25}{v}_{26}\}. \end{aligned}$$

#### Lemma 4

If ${G}_4$ is derived from the molecular structure of the anticancer medication Podophylotoxin, then ${Nul}\left( {G}_4\right) =0.$

#### Proof

It can be shown that ${G}_4$ represents the molecular structure of Podophylotoxin, an anticancer medication. This structure is characterized by the presence of pendant vertices, as well as a combination of two pentagons and three hexagons. By using the concept of nullity, we will ascertain the features polynomial ${CP}\left( {Bin}\left( {G}_4\right) ;{\lambda }\right) .$ The cost of the adjacency matrix, denoted as ${Bin}\left( {G}_4\right) ,$ is being considered. The graph ${G}_4$ is associated with a polynomial known as the characteristics polynomial.

$${CP}\left( {Bin}\left( {G}_4\right) ;{\lambda }\right) =$$

$$\begin{aligned}&{\lambda }^{30} - 34{\lambda }^{28} + 510{\lambda }^{26} - 4{\lambda }^{25} - 4461{\lambda }^{24} + 106{\lambda }^{23} + 25342{\lambda }^{22} - 1204{\lambda }^{21} - 98547{\lambda }^{20}\\ {}&+ 7714{\lambda }^{19} + 269335{\lambda }^{18} - 30878{\lambda }^{17} - 522716{\lambda }^{16} + 80788{\lambda }^{15} + 719038{\lambda }^{14} - 140428{\lambda }^{13}\\ {}&- 691984{\lambda }^{12} + 161650{\lambda }^{11} + 454416{\lambda }^{10} - 120784{\lambda }^9 - 195696{\lambda }^8 + 56402{\lambda }^7 + 51946{\lambda }^6\\ {}&- 15458{\lambda }^5 - 7615{\lambda }^4 + 2204{\lambda }^3 + 480{\lambda }^2 - 120{\lambda } - 4 \end{aligned}$$

By finding the solution to the equation ${CP}\left( {Bin}\left( {G}_4\right) ;{\lambda }\right) =0,$ we can determine the value of $\lambda .$For a given value of ${\lambda },$ the eigenvalues $\mathscr {E}\left( {CP}\left( {Bin}\left( {G}_4\right) ;{\lambda }\right) \right)$ of the specified polynomials are calculated.

$$\mathscr {E}\left( {CP}\left( {Bin}\left( {G}_4\right) ;{\lambda }\right) \right) =$$

$$\begin{aligned}&\{-2.5465, -2.3260, -2.0649, -1.9002, -1.7140, -1.6349, -1.6180, -1.4288, -1.2572,\\ {}&-1.0967, -0.7440, -0.6180, -0.5956, -0.3814, -0.0302, 0.2736, 0.5249, 0.6180, 0.6790,\\ {}&0.7984, 0.9332, 1.1578, 1.1995, 1.3662, 1.6180, 1.7592, 1.8341, 2.2556, 2.3443, 2.5947\}. \end{aligned}$$

It is observed that there is an absence of a precise zero value from the given data $\mathscr {E}\left( {CP}\left( {Bin}\left( {G}_4\right) ;{\lambda }\right) \right) =0$ and which is concluding that ${Nul}\left( {G}_4\right) =0.$

Moreover, Fig. 4 illustrates the presence of fifteen instances of undulating boundaries, which corresponds to the value of ${M}\left( {G}_4\right) .$

Furthermore, the total energy may be determined as 39.9131 by adding all the individual energies $\mathscr {E}\left( {CP}\left( {Bin}\left( {G}_4\right) ;{\lambda }\right) \right) .$

The determination has been made that the selected structure of the anticancer medicine is stable, consisting of closed-shell molecules, as shown by its nullity value of zero. $\square$

### The findings pertaining to the correlation between the number and nullity of the Pterocellin-B anticancer medication structure

The graph derived from Pterocellin-B has an order of 24, denoted as $|\mathscr {V}\left( {G}_{5}\right) |$, and size is 27, denoted as $|\mathscr {E}\left( {G}_{5}\right) |$. Furthermore, the node and lines sets have been established.

$$\begin{aligned} \mathscr {V}({G}_5)=&\{v_{i}:{i}=1,2,\dots ,24\},\\ {E}({{G}_5})=&\{{v}_{i}{v}_{{i}+1}:{i}=1,2,\dots ,14,18,19,\dots ,23\}\cup \{{v}_{1}{v}_{13},{v}_{1}{v}_{16},{v}_{3}{v}_{11},{v}_{4}{v}_{17},{v}_{5}{v}_{10},{v}_{9}{v}_{18},{v}_{19}{v}_{24}\}. \end{aligned}$$

#### Lemma 5

If ${G}_5$ is derived from the molecular structure of the anticancer medication Pterocellin-B, then ${Nul}\left( {G}_5\right) =0.$

#### Proof

It can be shown that ${G}_5$ represents the molecular structure of Pterocellin-B, an anticancer medication. This structure is characterized by the presence of pendant vertices, a single pentagon, and three hexagons. By using the concept of nullity, we will ascertain the features polynomial ${CP}\left( {Bin}\left( {G}_5\right) ;{\lambda }\right) .$ The cost of the adjacency matrix, denoted as ${Bin}\left( {G}_5\right)$, is being considered.The graph ${G}_5$ is associated with a characteristic polynomial.

$${CP}\left( {Bin}\left( {G}_5\right) ;{\lambda }\right) =$$

$$\begin{aligned}&{\lambda }^{24} - 27{\lambda }^{22} + 312{\lambda }^{20} - 2{\lambda }^{19} - 2030{\lambda }^{18} + 34{\lambda }^{17} + 8237{\lambda }^{16} - 238{\lambda }^{15} - 21822{\lambda }^{14} + 894{\lambda }^{13}\\ {}&+ 38406{\lambda }^{12} - 1968{\lambda }^{11} - 44759{\lambda }^{10} + 2594{\lambda }^9 + 33715{\lambda }^8 - 1992{\lambda }^7 - 15553{\lambda }^6 + 810{\lambda }^5 + 3933{\lambda }^4\\ {}&- 132{\lambda }^3 - 417{\lambda }^2 + 4 \end{aligned}$$

By finding the solution to the equation ${CP}\left( {Bin}\left( {G}_5\right) ;{\lambda }\right) =0$, we can determine the value of $\lambda$. For a given value of ${\lambda }$, the eigenvalues $\mathscr {E}\left( {CP}\left( {Bin}\left( {G}_5\right) ;{\lambda }\right) \right)$ of the specified polynomials are calculated.

$$\mathscr {E}\left( {CP}\left( {Bin}\left( {G}_5\right) ;{\lambda }\right) \right) =$$

$$\begin{aligned}&\{-2.3966, -2.2973, -2.0985, -1.8195, -1.4762, -1.3734, -1.2412, -1, -0.96824, -0.7397,\\ {}&-0.4769, -0.1050, 0.1012, 0.5698, 0.8605, 0.8898, 1, 1.1955, 1.3511, 1.4602, 1.7489, 2.0604,\\ {}&2.1899, 2.5653\}. \end{aligned}$$

It is observed that there is an absence of a precise zero value from the given data $\mathscr {E}\left( {CP}\left( {Bin}\left( {G}_5\right) ;{\lambda }\right) \right) =0$ and which is concluding that ${Nul}\left( {G}_5\right) =0.$

Moreover, Fig. 5 illustrates the presence of twelve instances of undulating boundaries, which corresponds to the value of ${M}\left( {G}_5\right) .$

Furthermore, the total energy may be determined as 31.9854 by adding all the individual energies $\mathscr {E}\left( {CP}\left( {Bin}\left( {G}_5\right) ;{\lambda }\right) \right) .$

The determination has been made that the selected structure of the anticancer medicine is stable, consisting of closed-shell molecules, as shown by its nullity value of zero. $\square$

### The findings pertaining to the correlation between the number and nullity of the Raloxifene anticancer medication structure

A network resulting from the administration of Raloxifene The cardinality of the vertex set of the graph ${G}_{6}$ is 34, but the cardinality of the edge set is 38. Furthermore, the node and lines set have been established by

$$\begin{aligned} \mathscr {V}({G}_6)=&\{v_{i}:{i}=1,2,\dots ,34\},\\ {E}({{G}_6})=&\{{v}_{i}{v}_{{i}+1}:{i}=1,2,\dots ,14,16,18,19,\dots ,31\}\cup \{{v}_{1}{v}_{6},{v}_{10}{v}_{15},{v}_{13}{v}_{16},{v}_{16}{v}_{18},\\ {}&{v}_{18}{v}_{26},{v}_{19}{v}_{24},{v}_{22}{v}_{34},{v}_{27}{v}_{32},{v}_{30}{v}_{33}\}. \end{aligned}$$

#### Lemma 6

If ${G}_6$ is derived from the molecular structure of the anticancer medication Raloxifene, then ${Nul}\left( {G}_6\right) =0.$

#### Proof

It may be shown that ${G}_6$ represents the molecular structure of Raloxifene, an anticancer medication. This structure consists of pendent vertices, a single pentagon, and four hexagons. By using the concept of nullity, we will ascertain the features polynomial ${CP}\left( {Bin}\left( {G}_6\right) ;{\lambda }\right) .$ The cost of the adjacency matrix of the graph denoted as ${Bin}\left( {G}_6\right)$ is being discussed.The graph ${G}_6$ is associated with a characteristic polynomial.

$${CP}\left( {Bin}\left( {G}_6\right) ;{\lambda }\right) =$$

$$\begin{aligned}&{\lambda }^{34} - 38{\lambda }^{32} + 650{\lambda }^{30} - 2{\lambda }^{29} - 6629{\lambda }^{28} + 58{\lambda }^{27} + 45015{\lambda }^{26} - 746{\lambda }^{25} - 215340{\lambda }^{24} + 5626{\lambda }^{23}\\ {}&+ 748987{\lambda }^{22} - 27724{\lambda }^{21} - 1927604{\lambda }^{20} + 94204{\lambda }^{19} + 3700145{\lambda }^{18} - 227028{\lambda }^{17} - 5298576{\lambda }^{16}\\ {}&+ 392980{\lambda }^{15} + 5617874{\lambda }^{14} - 488826{\lambda }^{13} - 4336919{\lambda }^{12} + 431794{\lambda }^{11} + 2366095{\lambda }^{10} - 263362{\lambda }^9\\ {}&- 866494{\lambda }^8 + 105010{\lambda }^7 + 193849{\lambda }^6 - 24504{\lambda }^5 - 21616{\lambda }^4 + 2520{\lambda }^3 + 600{\lambda }^2 \end{aligned}$$

The equation ${CP}\left( {Bin}\left( {G}_6\right) ;{\lambda }\right) =0$ may be solved. For a given value of ${\lambda }$, the eigenvalues $\mathscr {E}\left( {CP}\left( {Bin}\left( {G}_6\right) ;{\lambda }\right) \right)$ of the specified polynomials can be found.

$$\mathscr {E}\left( {CP}\left( {Bin}\left( {G}_6\right) ;{\lambda }\right) \right) =$$

$$\begin{aligned}&\{-2.3937, -2.2547, -2.2108, -2.1371, -1.9500, -1.6996, -1.5451, -1.3987, -1.2363,\\ {}&-1.0492, -1, -1, -1, -0.8551, -0.6549, -0.1316, -1.844e^{-15}, -1.2044e^{-15}, 0.3119, 0.6402,\\ {}&0.7840, 1, 1, 1, 1.0572, 1.2185, 1.3773, 1.4711, 1.7028, 1.8948, 2.1387, 2.1539, 2.2385, 2.527\}. \end{aligned}$$

It is observed that there is an absence of a precise zero value from the given data $\mathscr {E}\left( {CP}\left( {Bin}\left( {G}_6\right) ;{\lambda }\right) \right) =0$ and which is concluding that ${Nul}\left( {G}_6\right) =0.$

Moreover, Fig. 6 illustrates the presence of sixteen instances of undulating boundaries, which corresponds to the value of ${M}\left( {G}_6\right) .$

Furthermore, the total energy may be determined as 45.0338 by adding all the individual energies, denoted as $\mathscr {E}\left( {CP}\left( {Bin}\left( {G}_6\right) ;{\lambda }\right) \right) .$

The determination has been made that the selected structure of the anticancer medicine is stable, consisting of closed-shell molecules, as shown by its nullity value of zero. $\square$

## Discussion and conclusion

Several structures of anticancer drugs have been investigated, including Daunorubicin, Deguelin, Minocycline, Podophylotoxin, Pterocellin-B, and Raloxifene. The aforementioned structures are analyzed with respect to their characteristic, eigenvalues of the adjacency matrix polynomials, matching number and nullity. Consequently, the selected structures of the aforementioned anticancer drugs exhibit stability since they are composed of closed-shell molecules, characterized by a nullity value of zero. Furthermore, the findings are succinctly presented in Table 1.

**Table 1:** This study investigates the energy, positive-negative-inertia, and nullity properties of different structures of anticancer drugs.

*G*	*Nul*(*G*)	*p*(*G*)	*q*(*G*)	*E*
${G}_{1}$	0	19	18	48.1216
${G}_{2}$	0	14	17	39.8922
${G}_{3}$	0	18	18	41.4910
${G}_{4}$	0	15	15	39.9131
${G}_{5}$	0	12	12	31.9854
${G}_{6}$	0	16	18	45.0338

The formula for the first Betti number, denoted as ${b}_1(G)$, is given by the expression $\mathscr {M}+|C|-\mathscr {N}$, where $\mathscr {M}$ represents the number of edges in the graph, |*C*| denotes the count of graph components, and $\mathscr {N}$ represents a certain quantity. In the case when there is a single linked component, denoted as $|C|=1.$ The equation is modified to ${b}_1(G)=\mathscr {M}+1-\mathscr {N}$, where all selected drug structures are considered as linked components. The phrase “cyclomatic number,” which was developed by^61^. The cyclomatic number refers to the quantity of edge deletions required for a graph to transition into an acyclic state. In Elshoff’s study (1978), the concept of nullity is sometimes referred to as the cyclomatic number (*cn*(*G*)) or the first Betti number^62^. A comparison is made between the cyclomatic number of structures pertaining to anticancer drugs, first Betti number and the nullity, in the Table 2.

**Table 2:** There are many criteria that govern the characteristics of different architectures of anticancer drugs.

*G*	*Nul*(*G*)	${b}_1(G)$	*cn*(*G*)
${G}_{1}$	0	5	5
${G}_{2}$	0	5	5
${G}_{3}$	0	4	4
${G}_{4}$	0	5	5
${G}_{5}$	0	4	4
${G}_{6}$	0	5	5

Based on the comparison shown in Table 2, it can be seen that the initial Betti number and nullity of the various structures of anticancer drugs exhibit dissimilarities. Remarkably, it has been shown that the cyclomatic number and the first Betti number exhibit identical values across all configurations.

## Data Availability

The datasets used and/or analysed during the current study available from the corresponding author on reasonable request.
